# Changes in expression of p53 and inflammatory factors in patients with ulcerative colitis

**DOI:** 10.3892/etm.2019.7994

**Published:** 2019-09-10

**Authors:** Hui Su, Qian Kang, Haihong Wang, Hui Yin, Linghui Duan, Yuli Liu, Ruying Fan

Exp Ther Med 17: 2451-2456, 2019; DOI: 10.3892/etm.2019.7253

Following the publication of the above article, the authors have noticed that the acceptance date for the paper was printed incorrectly: “November 19, 2019” should have appeared as “November 19, 2018”. The Editor apologizes to the authors for this error, and for the inconvenience caused.

